# A Novel Suturing Technique for Choroidal Avulsion

**DOI:** 10.3390/jcm11185344

**Published:** 2022-09-12

**Authors:** Takeshi Iwase, Shungo Nishiyama, Mariko Sato

**Affiliations:** Department of Ophthalmology, Akita University Graduate School of Medicine, Akita 010-8543, Japan

**Keywords:** choroidal avulsion, globe rupture, suturing technique, 10-0 proline, 7-0 nylon

## Abstract

Ocular trauma has been one of the leading causes of visual impairment, and choroidal avulsion is especially devastating. Surgical treatment of choroidal avulsion is challenging, and very few surgical techniques have been reported. We experienced two cases of globe rupture with 360-degree avulsion of the choroid-ciliary body from the peripheral section. After vitrectomy for a globe rupture, the choroid gradually slid down to the posterior pole over time and vision deteriorated even though the retina was attached. We treated the choroidal avulsion using two surgical methods: a mattress suturing technique using a 10-0 proline long needle and a 7-0 nylon single suture technique. In both methods, the retina-choroid, which had slipped down to the posterior pole, was suspended and fixed to the sclera assisted by a wide-angle viewing system, improving visual acuity. These two methods are considered to be useful surgical procedures for the treatment of an avulsed choroid.

## 1. Introduction

Ocular trauma is one of the leading causes of visual impairment worldwide, especially when the posterior segment of the eye is involved [1,2]. Traumatic choroidal injury is a serious complication and is closely related to the prognosis of ocular trauma, and a variety of choroidal injuries have been reported [3,4,5,6]. It has been reported that choroidal avulsion is especially devastating, with 92.2% of choroidal avulsion cases having a poor prognosis [7].

Choroidal avulsion is the detachment of the choroid from the sclera, with discontinuity of the detached choroid, or sometimes with the choroid and ciliary body together at the scleral spur as a whole unit [7]. However, few reports have focused on choroidal avulsion, mainly because of the lack of effective treatment. Even with the development of vitreoretinal surgery techniques, there are very few treatments for choroidal avulsion [3,5].

Surgical treatment of choroidal avulsion is complicated and very challenging. In this study, we treated two cases with choroidal avulsion, in which the retina-choroid had slipped down to the posterior pole, using two different surgical techniques assisted by a wide-angle viewing system. These two methods are considered to be useful surgical procedures for the treatment of an avulsed choroid.

## 2. Patients and Methods

The ethics committee of Akita University Hospital (Akita, Japan) approved the procedures, and the procedures conformed to the tenets of the Declaration of Helsinki. Informed consent was obtained from participants after explaining the nature and possible complications of the study.

### 2.1. Subjects

**Figure 1:**
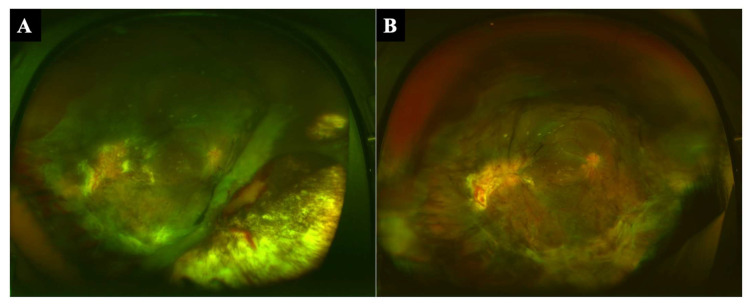
Fundus photographs before and after the mattress suturing technique. A 17-year-old man had choroidal avulsion after surgery for globe rupture. The retina-choroid gradually slid down together to the posterior pole, and the exposed sclera gradually widened, especially in the infero-nasal quadrant (**A**). The retina-choroid, which had been sliding down to the posterior pole, was stretched after the repair of the choroidal avulsion (**B**).

Patient 1: A 17-year-old man had globe rupture, vitreous hemorrhage, retinal detachment, and choroidal hemorrhage, and his vision was limited to light perception in 2017. We performed vitrectomy with concomitant encircling. The lens was not present in the eye, and a 360-degree choroidal-ciliary body avulsion was observed during surgery. Silicone oil was injected into the vitreous cavity after drainage of the choroidal hemorrhage from the sclera, fluid–air exchange, and laser photocoagulation. The retina was attached, and visual acuity improved to 20/150. Although the retina remained attached, the retina-choroid gradually slid down to the posterior pole together, and part of the choroid was rolling (Figure 1A). The exposed sclera gradually widened, and the retina-choroid was folded at the posterior pole. The patient was aware of visual field narrowing, and visual acuity decreased to 20/2000 four months after the surgery. The intraocular pressure (IOP) was 3 mmHg. Choroidal suture was then scheduled to repair the choroidal avulsion.

**Figure 2:**
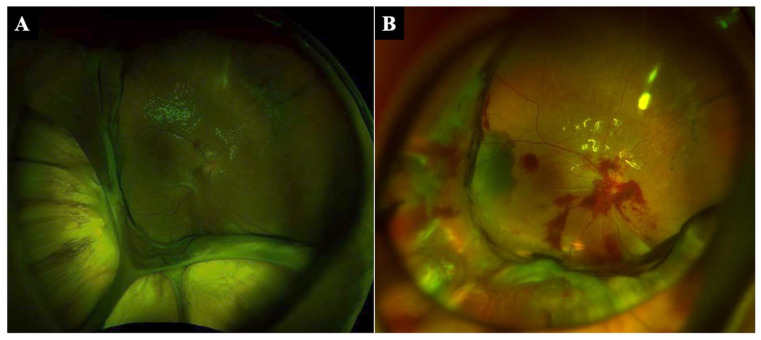
Fundus photographs before and after the 7-0 nylon single suture technique. A 55-year-old woman underwent scleral suturing and vitrectomy for globe rupture, and it was found that the choroid-ciliary body was avulsed at 360 degrees during surgery. The choroid was still detached from the sclera postoperatively, the retina-choroid gradually slid downward to the posterior pole together, and the exposed sclera widened with time (**A**). The avulsed choroid was sutured to the sclera, and the exposed sclera could be covered by the retina-choroid (**B**).

Patient 2: A 55-year-old woman had globe rupture, vitreous hemorrhage, retinal detachment, and severe choroidal hemorrhage, and her vision was limited to light perception in 2020. During initial surgery with scleral suturing and vitrectomy, it was found that the lens was not present in the eye and that the choroid-ciliary body was avulsed at 360 degrees. After drainage of the choroidal hemorrhage from the sclera, fluid–air exchange, and silicone oil tamponade, the retina was successfully attached, and her visual acuity improved to 20/250. However, the choroid was still detached from the sclera postoperatively, and the retina-choroid gradually slid downward to the posterior pole together and the exposed sclera widened with time (Figure 2A). Visual acuity decreased to 20/1000 three months after the surgery, and the IOP was 4 mmHg. The patient underwent additional surgery for choroidal suturing for choroidal avulsion.

### 2.2. Surgical Techniques

Patients with choroidal avulsion received a 25-gauge pars plana vitrectomy under retrobulbar anesthesia. After creating the three ports, two different suturing techniques to reattach the avulsed choroid to the sclera were performed on the patients with an avulsed choroid. In both suturing techniques, a wide-angle viewing system and chandelier illumination was used to assist bimanual manipulation.

**Figure 3:**
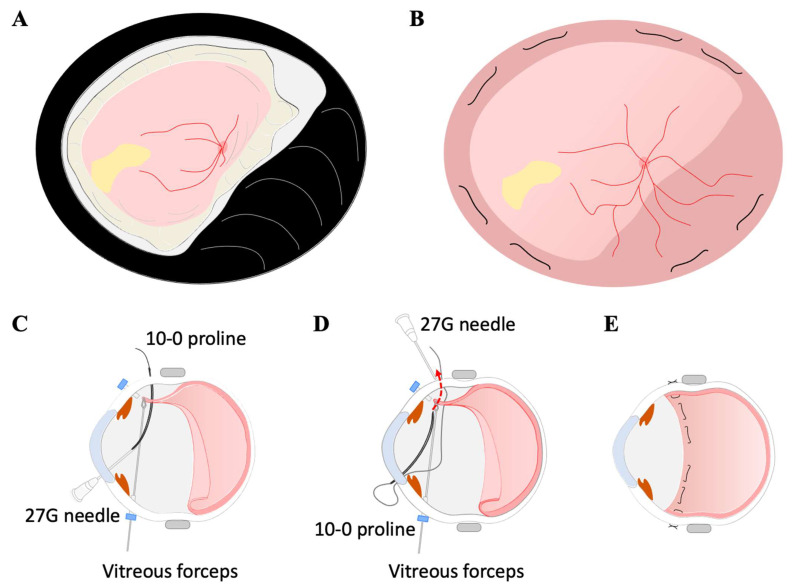
Surgical procedures of the mattress suturing technique using a 10-0 proline long needle. Fundus schema before (**A**) and after the repair of the choroidal avulsion (**B**). A 10-0 proline needle was inserted into the vitreous cavity and passed through the avulsed choroid by grasping the avulsed choroidal edge with vitreous forceps (**C**). A 27G needle was inserted through the corneal side port to access the vitreous body, and the proline needle was inserted into the 27G needle intravitreously. After the proline needle was withdrawn from the 27G needle, the 10-0 proline was inserted through the same corneal side port, and the 27G needle was inserted into the vitreous cavity through the sclera (**D**). The 27G needle was passed through the avulsed choroid by grasping the avulsed choroidal edge with vitreous forceps. The same procedure was performed at two locations in each quadrant (**E**).

For the mattress suturing technique, a 10-0 proline long needle (1713G, ETHICON, LLC, San Lorenzo, PR, USA) in an aphakic eye was inserted into the vitreous cavity at the sclera 6 mm posterior to the limbus (Figure 3). It was passed through the avulsed choroid by grasping the avulsed choroidal edge with vitreous forceps (V-ARTIST disposable micro forceps, HOYA Corporation, Tokyo, Japan) (Figure 3C). Because the eye was aphakic, a 27G needle was inserted through the corneal side port to access the vitreous body, and the proline needle was inserted into the 27G needle intravitreously. The 27G needle with the proline needle was withdrawn from the side port in the cornea. After the proline needle was withdrawn from the 27G needle, the 10-0 proline was inserted through the same corneal side port and retained in the anterior chamber, and the 27G needle was inserted into the vitreous cavity through the sclera approximately 5 mm apart horizontally from the insertion site of the sclera. The 27G needle was passed through the avulsed choroid by grasping the avulsed choroidal edge with vitreous forceps. The proline needle was inserted into the 27G needle intravitreously, as before, and the 27G needle with the proline needle was withdrawn from the sclera. The 10-0 proline thread was sutured outside the sclera. The same procedure was performed at two locations in each quadrant, and the avulsed choroid could be sutured to the sclera. During the procedure, the choroid was torn several times due to the mattress suturing attempts to secure the choroid as much as possible to the peripheral sclera. However, no choroidal hemorrhage occurred when the choroid was torn.

**Figure 4:**
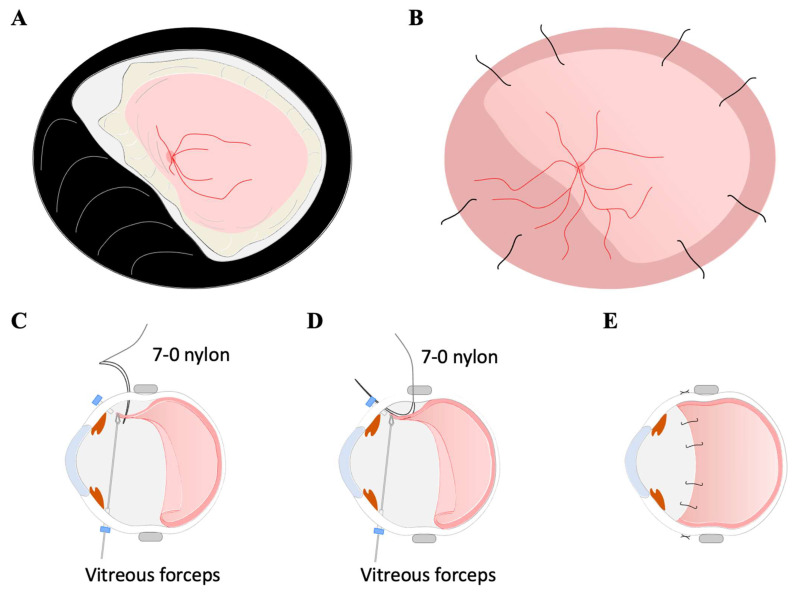
Surgical procedures of the 7-0 nylon single suture technique. Fundus schema before (**A**) and after the repair of the choroidal avulsion (**B**). A 7-0 nylon was introduced into the vitreous cavity at the sclera 6 mm posterior to the limbus (**C**). It was passed through the avulsed choroid by grasping the anterior margin of the avulsed full-thickness choroid to accomplish suturing. The needle was then rotated vertically, and the 7-0 nylon was withdrawn from the sclera at the position of the ciliary body, trying to suture the choroid to the sclera as peripherally as possible (**D**). The 7-0 nylon needle was sutured outside the sclera. The same procedure was performed at two locations in each quadrant (**E**).

For the 7-0 nylon single suture technique, a 7-0 nylon (1696G, ETHICON, LLC, San Lorenzo, PR, USA) was introduced into the vitreous cavity at the sclera 6 mm posterior to the limbus (Figure 4). It was passed through the avulsed choroid by grasping the anterior margin of the avulsed full-thickness choroid to accomplish suturing. The needle was then rotated vertically, and the 7-0 nylon was withdrawn from the sclera at the position of the ciliary body, trying to suture the choroid to the sclera as peripherally as possible. At that time, an assistant grasped the tip of the 7-0 nylon needle as it emerged from the sclera, taking care that the 7-0 nylon needle did not stray into the vitreous cavity. The 7-0 nylon needle was sutured outside the sclera. The same procedure was performed at two locations in each quadrant.

Finally, fluid–air exchange and silicone oil tamponade were performed at the end of the surgery in both surgical procedures.

## 3. Results

Patient 1: The retina-choroid, which had slipped down to the posterior pole, was suspended and fixed to the sclera after the mattress suturing technique using a 10-0 proline long needle (Figure 1B). The mattress suturing methods allowed the choroid to be sutured over a wide area and close to its original position in the periphery. The folded retina of the posterior pole was stretched, and vision was improved to 20/200. The subjective symptoms of a narrowing of the visual field also improved. However, the IOP was still 3 mmHg after the surgery. 

Patient 2: The retina-choroid was fixed to the sclera, and the exposed sclera was mostly covered with retina-choroid after the 7-0 nylon single suture technique (Figure 2B). The retina-choroid, which had slid downward to the posterior pole, was stretched, and vision was improved to 20/250. The IOP was 8 mmHg after the surgery.

## 4. Discussion

In this study, we treated choroidal avulsion using two surgical methods. In both methods, the retina-choroid, which had slipped down to the posterior pole, was suspended and fixed to the sclera assisted by a wide-angle viewing system, unfolding the retina in the posterior pole, and improving visual acuity. These two methods are considered to be useful surgical procedures for the treatment of an avulsed choroid.

Choroidal avulsion is one of the most serious traumatic conditions [1,2]. If the choroid and ciliary body are disrupted as a whole unit at the scleral spur and lose their adhesion to the sclera, the choroid in that region is easily detached. When the choroid-ciliary disruption at the scleral spur is 360 degrees circumferentially, the choroid slides down to the posterior pole. Because of the different tissue composition of the retina and choroid, it is difficult to reattach the avulsed choroid by the usual methods used in the treatment of retinal detachment. 

Despite the development and improvement of microsurgical techniques in vitreoretinal surgery, eyes with choroidal avulsion can rarely be repaired. This is due to the connection between the vitreous cavity and the suprachoroidal space after uveal injury, which results in a free flow of aqueous humor between these two compartments and a decrease in intraocular pressure. In addition, the formation of these two compartments significantly reduces the volume of the vitreous cavity, thus reducing the likelihood of retinal reattachment. Thus, without effective treatment, eyes with choroidal detachment usually suffer from phthisis bulbi and cannot be saved.

Very few surgical treatments have been attempted to repair choroidal avulsion. It has been reported that medical fibrin glue was injected into the suprachoroidal space before silicone oil injection [5]. However, because fibrin glue can only be applied after fluid–air exchange in a non-water surgical environment, its use has been limited in many cases to repairing an avulsed choroid before the retina is reattached. A method in which a full-thickness scleral incision of the same length as an extension of the avulsed area is made at the equator, and the avulsed choroid is incarcerated into the scleral incision and sutured together has been reported [3]. However, this suturing technique may result in exposure of the uvea and may cause further sympathetic ophthalmopathy. 

When using the 10-0 proline procedure, we grasped the anterior margin of the avulsed choroid to accomplish suturing with vitreous forceps and passed the proline needle through to perform mattress suturing at the ideal width. Mattress suturing methods allow the choroid to be sutured over a wide area and close to its original position in the periphery. A major problem in suturing the choroid is that the choroid itself is a fragile tissue that tears easily when strong traction is applied. In other words, the choroid is easily torn if it is forcibly suspended in the periphery using mattress suturing methods when there is not enough choroid-ciliary body area to completely cover the exposed sclera. Therefore, although we would prefer to perform mattress suturing in a more peripheral area, it is important to perform this technique in each quadrant with some length to spare.

The 7-0 nylon needle is long and curved, making it suitable for threading the sclera, choroid, and sclera in a single vertical operation. Suturing with a 7-0 nylon needle is relatively simple, allowing multiple threads to be sutured in a short time. In the present case, the suture was made vertically, so a U-turn suture was not used. However, it is considered possible to perform a U-turn suture by suturing in the circumferential direction. Considering that a U-turn suture using a 10-0 proline needle procedure can be performed only in aphakic eyes and that the procedure is complicated, we believe that a circumferential suture using a 7-0 nylon needle may be useful for U-turn suturing in the future.

A possible complication of choroidal suturing is choroidal hemorrhage. The choroid is a highly vascularized tissue, and bleeding occurs not only from trauma but also from many other causes. The blood circulation in the choroid should be severely affected after choroidal avulsion occurs; therefore, suturing the avulsed choroid seldom causes bleeding. In the present procedure, there was no bleeding when a 10-0 proline was used, and some bleeding occurred when a 7-0 nylon needle was used, but it was controllable with the endo-diathermy.

## 5. Conclusions

In this study, we treated choroidal avulsion using two surgical methods. In both methods, the retina-choroid, which had slipped down to the posterior pole, was suspended and fixed to the sclera assisted by a wide-angle viewing system, unfolding the retina in the posterior pole, and improving visual acuity. These two methods are considered to be useful surgical procedures for the treatment of an avulsed choroid.

## Data Availability

The data that support the findings of this study are available from the corresponding author upon reasonable request.

## Abbreviation

The following abbreviation is used in this manuscript:

IOP intraocular pressure
